# Enhanced Antimicrobial Activity of Green-Synthesized *Artemisia*-ZnO Nanoparticles: A Comparative Study with Pure ZnO Nanoparticles and Plant Extract

**DOI:** 10.3390/foods14142449

**Published:** 2025-07-11

**Authors:** Noor Akhras, Abuzer Çelekli, Hüseyin Bozkurt

**Affiliations:** 1 Department of Biochemistry Science and Technology, Faculty of Arts and Science, Gaziantep University, Gaziantep 27310, Türkiye; nurak.1811@gmail.com; 2 Department of Biology, Faculty of Art and Science, Gaziantep University, Gaziantep 27310, Türkiye; celekli.a@gmail.com; 3 Department of Food Engineering, Faculty of Engineering, Gaziantep University, Gaziantep 27310, Türkiye

**Keywords:** zinc oxide nanoparticles (ZnO NPs), *Artemisia absinthium* L. extract, eco-friendly process, characterization, antimicrobial activity

## Abstract

The green synthesis of zinc oxide nanoparticles (ZnO NPs) using *Artemisia absinthium* L. extract has gained considerable attention due to its eco-friendly approach and potential applications in food science. This study investigates the synthesis and characterization of *Artemisia*-mediated ZnO NPs, focusing on their physicochemical properties. The nanoparticles were characterized using ultraviolet–visible spectroscopy (UV–Vis), Fourier transform infrared spectroscopy (FT-IR), field emission scanning electron microscopy (FE-SEM), and energy dispersive X-ray spectroscopy (EDX). Successful synthesis was achieved through a co-precipitation method, resulting in an average particle size of 36.6 nm. The presence of polyphenols and flavonoids in *A. absinthium* L. extract acted as both a reducing agent and stabilizer for the nanoparticles. The physicochemical characterization revealed strong absorption peaks indicative of ZnO, confirming successful nanoparticle formation. In addition to the structural findings, this study presents novel insights by demonstrating that *Artemisia*-mediated ZnO NPs possess significantly enhanced antimicrobial activity compared to both pure ZnO NPs and the plant extract alone. The biosynthesized nanoparticles exhibited notably lower minimum inhibitory concentration (MIC) and minimum bactericidal/fungicidal concentration (MBC/MFC) values against *Staphylococcus aureus*, *Escherichia coli*, and *Candida albicans*, suggesting a strong synergistic effect between ZnO and the phytochemicals of *A. absinthium* L. Thus, the study confirms and quantifies the superior antibacterial potential of *Artemisia*-derived ZnO NPs, offering promising implications for food, biomedical and pharmaceutical applications.

## 1. Introduction

The increasing global demand for environmentally sustainable and biologically safe nanomaterials has led to growing attention to green synthesis methods for producing metal oxide nanoparticles. Among them, zinc oxide nanoparticles (ZnO NPs) have attracted significant interest due to their unique physicochemical and biological properties, such as high surface area, semiconductor characteristics, and notable antimicrobial, antioxidant, and anticancer activities. These properties make ZnO NPs highly versatile for applications in biomedicine, food packaging, pharmaceuticals, and cosmetics [1,2,3]. Conventional physical and chemical synthesis methods, such as sol–gel, hydrothermal, microwave-assisted, and chemical precipitation techniques, typically require high temperatures, organic solvents, or hazardous precursors [1,4]. These methods often involve hazardous chemicals and generate toxic by-products, raising concerns about environmental and health problems. Therefore, a growing need has emerged for alternative methods that align with the principles of green chemistry [4]. Among the most promising approaches is the green synthesis of ZnO NPs using biological systems such as plants, fungi, algae, and bacteria. Plant-mediated synthesis is particularly attractive due to its simplicity, cost-effectiveness, potential for large-scale production, and the multifunctional role of plant-derived metabolites, which act as reducing, capping, and stabilizing agents [5]. In recent years, numerous studies have demonstrated the successful synthesis of ZnO NPs using plant extracts, highlighting the advantages of green synthesis in terms of particle size control, surface functionalization, and enhanced biological activity [6,7,8,9,10]. For instance, ZnO NPs synthesized by using *Artemisia* plant extract demonstrated notable anti-biofilm activity, along with desirable physicochemical properties, emphasizing their potential in biomedical applications [4]. However, despite the growing number of reports on green ZnO synthesis, many studies still lack comprehensive physicochemical characterization or comparative assessments of biological efficacy compared to conventional synthesis.

*Artemisia absinthium* L. (commonly known as wormwood), a plant rich in bioactive compounds such as flavonoids, terpenoids, and polyphenols, has been identified as a suitable candidate for the green synthesis of ZnO NPs. *A. absinthium* L. exhibits anti-inflammatory, anti-cancer, antioxidant, and other health-promoting properties. Additionally, it is rich in flavonoids and terpenoids, with over 160 flavonoid compounds [6]. *A. absinthium* L. has traditionally been used as a febrifuge, antiseptic, antihelminthic, tonic, and diuretic. The genus *Artemisia*, consisting of small herbaceous plants, is one of the largest and most widely distributed genera in the *Asteraceae* family [7]. In Turkish flora, there are approximately 22 species of *Artemisia*. Camphor, one of the key components of *A. absinthium* L., constitutes 1.4% of its essential oil [7]. The gastroprotective effects of *A. absinthium* L. have been confirmed using its water-soluble extract at room temperature, particularly in the aerial parts of the plant [8]. Additionally, it has been demonstrated that the addition of *A. absinthium* L. to food is safe and enriches food products with beneficial substances [9]. *A. absinthium* L. is widely utilized not only in medicine but also in the food industry and is believed to have appetite-stimulating, digestive, and tonic effects [10]. Many studies have reported on the antimicrobial activities of *Artemisia* species. Its extract exhibits antioxidant and antimicrobial effects, supporting its use as an adjunctive therapy [11]. In the cosmetics industry, *A. absinthium* L. is also incorporated into various products due to its strong antimicrobial and antioxidant properties [10].

*Artemisia absinthium* L. stands out among medicinal plants for nanoparticle synthesis due to its concentrated and multifunctional phytochemicals, which play key roles in the reduction, capping, and stabilization of ZnO nanoparticles. Flavonoids—such as quercetin, apigenin, and luteolin—act as natural reducing agents by donating electrons to Zn^2+^ ions, thereby facilitating the formation of ZnO nuclei. These compounds also bind to the nanoparticle surface through their hydroxyl and carbonyl groups, functioning as stabilizing agents that prevent agglomeration and enhance colloidal stability [6]. Terpenoids and sesquiterpene lactones, abundant in *A. absinthium* L., contribute further by interacting with metal ions and directing anisotropic growth, influencing particle morphology and crystal orientation [6,7]. The presence of camphor, a volatile monoterpene component of the essential oil, adds to the plant’s reducing potential and may assist in controlling particle dispersion. Compared to other plant species used in green synthesis, *Artemisia* species are particularly rich in multifunctional compounds with antioxidant, antimicrobial, and anti-inflammatory effects, which can enhance the biological activity of the resulting nanoparticles [7,8]. This biochemical richness gives *A. absinthium* L. a dual advantage; it not only enables efficient nanoparticle synthesis but also imparts bioactivity to the particles, making them more effective for applications in biomedicine, food preservation, and cosmetics [12,13].

Notably, *A. absinthium* L. has been employed in green synthesis studies to fabricate nanoparticles, demonstrating promising antimicrobial and antioxidant properties [4,5,12]. However, there is a lack of comprehensive studies investigating its efficacy in synthesizing ZnO NPs and comparing the resulting nanomaterials with chemically synthesized ZnO NPs or the plant extract alone. In this context, the present study aims to fill this gap by synthesizing ZnO nanoparticles using an aqueous extract of *A. absinthium* L. via a green co-precipitation method. The nanoparticles were characterized using UV–Vis spectroscopy, FT-IR, FE-SEM, and EDX spectroscopy to evaluate their size, morphology, surface chemistry, and elemental composition. Moreover, the antimicrobial activity of the green-synthesized ZnO NPs was assessed and compared with that of pure ZnO NPs and the plant extract against *Staphylococcus aureus*, *Escherichia coli*, and *Candida albicans*. To the best of our knowledge, this is one of the first studies that not only utilizes *A. absinthium* L. for the green synthesis of ZnO NPs but also compares their antimicrobial efficacy with that of conventionally synthesized ZnO NPs and the crude plant extract. This comparative framework allows for the evaluation of synergistic effects and highlights the added value of green synthesis beyond environmental sustainability. By addressing the limitations of previous studies and integrating a systematic review of the literature, this work advances the understanding of phytochemical-assisted nanomaterial design and contributes to the development of future biomedical and food safety applications.

## 2. Materials and Methods

### 2.1. Materials and Reagents

Folin–Ciocalteu phenol reagent, gallic acid, sodium carbonate (Na_2_CO_3_), aluminum chloride, 1,1-diphenyl-2-picrylhydrazyl (DPPH), and potassium acetate were obtained from Sigma-Aldrich (Darmstadt, Germany). Standard phenolic and flavonoid compounds, including chlorogenic acid, caffeic acid, p-coumaric acid, kaempferol, apigenin, pinocembrin, artepillin C, galangin, 2,4-dimethoxycinnamic acid, trans-ferulic acid, gallic acid, procyanidin B2, quercetin, and catechin, were also purchased from Sigma-Aldrich (Darmstadt, Germany). Zinc nitrate hexahydrate, sodium hydroxide (NaOH), ethanol (99.9%), and methanol (99.9%) were purchased from Merck (Darmstadt, Germany). Petri dishes were supplied by ISOLAB (Wertheim, Germany). Mueller–Hinton agar, Mueller–Hinton broth, Sabouraud dextrose agar, and antibiotic discs, including tetracycline (30 μg) and Fluconazole (25 μg), were obtained from Oxoid Ltd. (Hampshire, UK). The 0.5 McFarland turbidity standard was purchased from Thermo Fisher Scientific (Waltham, MA, USA).

### 2.2. Methods

#### 2.2.1. Plant Collection and Extraction

The *A. absinthium* L. plants used in this study were collected from Gaziantep province in Türkiye. Botanical authentication was performed by the Department of Biology and Food Engineering at Gaziantep University. The plant materials were initially air-dried, followed by shade-drying to ensure the removal of residual moisture. Subsequently, the dried samples were ground into a fine powder and stored in airtight glass containers under appropriate conditions. The powdered sample was then subjected to Soxhlet extraction for further analysis.

#### 2.2.2. Extraction Process (Gradient Program)

The chemical profile of *A. absinthium* L. was investigated through high-performance liquid chromatography (HPLC), by using thirteen standard polyphenol compounds: chlorogenic acid, caffeic acid, p-coumaric acid, kaempferol, apigenin, pinocembrin, artepillin, galangin, 2,4-dimethoxycinnamic acid, trans-ferulic acid, gallic acid, procyanidin B2, quercetin, and catechin (Sigma-Aldrich, Waltham, MA, USA). The identified compounds were compared with reference standards. Next, 5 g of the standard dry powder was measured into a 100 mL flask, and 50 mL of pure methanol was added, followed by 15–30 s of shaking. Then, 45 mL of deionized water was added, shaken again, and then adjusted to the final volume with deionized water. Next, 20–30 mL of the suspension was filtered through filter paper, with the first 10 mL of filtrate discarded. The remaining filtrate was drawn into a syringe and passed through a 0.45 µm filter into vials [14].

#### 2.2.3. The Synthesis of *Artemisia*-Zinc Oxide Nanoparticles (*Artemisia*-ZnO NPs)

A total of 10 g of the powdered plant was combined with 200 mL of deionized water and heated to 80 °C on a heater–stirrer for one hour. After heating, the mixture was filtered and centrifuged at 15,000 rpm for 15 min to eliminate plant residues and impurities. The resulting extract was then stored for future use. The ZnO NPs were synthesized using a co-precipitation method based on the previously described procedure [15], with some modification. For the green synthesis of *Artemisia*-ZnO NPs, 25 mL of a 0.05 M Zn(NO_3_)_2_·6H_2_O solution was mixed with 10 mL of *Artemisia* aqueous extract. The mixture was stirred for one hour at 80 °C using a magnetic stirrer. To adjust the pH between 8 and 12, 0.2 M NaOH was added drop by drop. The mixture was then stirred for an additional hour, resulting in the formation of a solid product. The precipitate was purified by multiple re-dispersions in deionized water at 15,000 rpm for 15 min (EBA 20. Hettich, Kirchlengern, Germany), followed by final washings with ethanol. After centrifugation, the product was incubated at 80 °C overnight. The dried powder was hand-ground into a fine powder to get the pure *Artemisia*-ZnO NPs. The same procedure was applied to prepare the pure zinc oxide nanoparticles ZnO NPs (as a control) without using the extract of *A. absinthium* L.

#### 2.2.4. Characterization of *Artemisia*-ZnO NPs

The biosynthesized *Artemisia*-ZnO NPs were characterized using various analytical techniques. Prior to analysis, the ZnO nanoparticles were thoroughly washed with distilled water and ethanol to remove unreacted biological residues, followed by centrifugation at 8000 rpm for 15 min. The purified samples were then oven-dried at 60 °C for 12 h and stored in airtight containers for further use. For UV–Vis spectroscopy, 1 mL of the aqueous suspension of purified ZnO NPs was redispersed in distilled water and sonicated at 4000 rpm for 15 min to ensure homogeneity. The absorbance spectrum was recorded in the range of 200–400 nm using a UV-1800 spectrophotometer (Shimadzu, Tokyo, Japan). For FT-IR analysis, the dried ZnO nanopowder was finely ground with potassium bromide (KBr) to form a pellet and analyzed in the range of 4000–400 cm^−1^ using a Perkin Elmer 100 Spectrum spectrometer (Berlin, Germany) to identify functional groups and confirm phytochemical interactions. For FE-SEM imaging, a small amount of the dried nanoparticle powder was mounted on carbon tape over an aluminum stub and coated with a thin layer of gold using a sputter coater to improve conductivity. Morphological features were observed using a Zeiss SIGMA VP-500 field emission scanning electron microscope (Berlin, Germany). For EDX analysis, the same FE-SEM prepared sample was used to determine elemental composition using an Oxford Instruments EDX detector (Oxford, UK). All characterizations were performed in triplicate to ensure the reliability of results.

#### 2.2.5. Sample Preparation for HPLC Phenolic Compound Analysis

The phenolic compound profile of *A. absinthium* L. extract was analyzed using high-performance liquid chromatography (HPLC) following a previously described method [14], with slight modifications. The extract was first centrifuged at 5000 rpm for 3 min to remove particulates. A 100 mL of the supernatant was filtered through a 0.45 µm PTFE syringe filter (Sigma-Aldrich, USA) to ensure clarity and compatibility with the HPLC system. From this filtered solution, 25 µL was injected into the HPLC system (Shimadzu SPD-20A, Japan) equipped with a C18 analytical column (4.6 mm × 250 mm, 5 µm; GL Sciences, Tokyo, Japan). The separation was performed using a two-solvent gradient elution system: solvent A (2:98, *v*/*v* acetic acid–deionized water) and solvent B (50:50, *v*/*v* methanol–deionized water). The gradient program was as follows: 3% B at 0 min, 5% at 3 min, 20% at 20 min, 25% at 30 min, 30% at 40 min, 50% at 55 min, and 100% B at 65 min, which was maintained for 10 min before returning to initial conditions. The flow rate was set at 1 mL/min, the column temperature was maintained at 30 °C, and phenolic compounds were detected at 280 nm based on their UV absorption spectra. Quantification was performed using external calibration curves of known phenolic standards, and results were expressed in mg/L (ppm). All analyses were conducted in triplicate.

#### 2.2.6. Determination of Total Phenolic Content (TPC), Total Flavonoid Content (TFC), and Antioxidant Activity (DPPH)

##### Preparation of *Artemisia*-ZnO NPs Suspension

To analyze the phytochemical content and antioxidant activity, 10 mg of ZnO NPs synthesized using *A. absinthium* L. extract at 80 °C were dispersed in 10 mL of methanol and sonicated for 30 min at room temperature. The suspension was then centrifuged at 5000 rpm for 10 min, and the supernatant was collected for analysis to minimize interference from the inorganic matrix.

##### Total Phenolic Content (TPC)

TPC of *A. absinthium* L. extract was measured using the Folin–Ciocalteu method [16]. A total of 450 μL of the extract was combined with 2.25 mL of Folin–Ciocalteu reagent, which had been pre-diluted with distilled water in a 1:9 ratio. After shaking for 3 min at room temperature, 1.8 mL of a sodium carbonate solution (75 g/L) was added. The mixture was then allowed to react in a dark environment at room temperature for 2 h. The phenolic content was analyzed spectrophotometrically (Optima SP 3000 Nano, Tokyo, Japan) at 760 nm. To create a calibration curve, 450 μL of gallic acid solution (10–80 μg/mL) was processed under the same conditions. The TPC values were expressed as mg gallic acid equivalents (GAE) per gram of *A. absinthium* L. All measurements were performed in triplicate to ensure accuracy. 

TPC of *Artemisia*-ZnO NPs was also determined using the Folin–Ciocalteu method. Briefly, 200 µL of the nanoparticle extract supernatant was mixed with 1.0 mL of 10% Folin–Ciocalteu reagent and incubated for 5 min. Then, 800 µL of 7.5% sodium carbonate solution was added, and the mixture was incubated in the dark at room temperature for 30 min. The absorbance was measured at 760 nm using a UV–Vis spectrophotometer. Gallic acid was used to prepare the calibration curve, and results were expressed as mg gallic acid equivalent per gram of ZnO sample (mg GAE/g).

##### Determination of Total Flavonoid Content (TFC)

TFC of the extracts was determined using the aluminum chloride colorimetric method [16]. A calibration curve was established using quercetin, where 10 mg of quercetin was dissolved in 80% methanol and then diluted to concentrations of 10, 25, 50, and 100 µg/mL. Each 0.5 mL of these solutions was separately mixed with 1.5 mL of 95% ethanol, 0.1 mL of 10% aluminum chloride, 0.1 mL of 1 M potassium acetate, and 2.8 mL of distilled water. The mixtures were incubated at room temperature for one hour, after which their absorbance was measured at 415 nm using a spectrophotometer (Optima SP 3000 nano, Japan). The same procedure was applied to 0.5 mL of the ethanol extract sample. The TFC content was expressed as milligrams of quercetin equivalents per gram of *A. absinthium* L. (mg QE/g).

TFC of *Artemisia*-ZnO NPs was measured using the same method described previously [16]. A 500 µL aliquot of the nanoparticle supernatant was mixed with 1.5 mL of methanol, 100 µL of 10% aluminum chloride, 100 µL of 1 M potassium acetate, and 2.8 mL of distilled water. After incubation at room temperature for 30 min, the absorbance was recorded at 415 nm. Quercetin was used as the standard, and TFC was expressed as mg quercetin equivalent per gram of ZnO NPs sample (mg QE/g).

##### Determination of Diphenylpicrylhydrazyl (DPPH) Radical Scavenging Activity

The DPPH scavenging activity of the samples was assessed following the method described in [16]. A 2500 µL methanolic DPPH solution (89.7 µmol/L, adjusted to a final absorbance of 0.800 ± 0.010 AU at 517 nm) was mixed with 500 µL of either the sample extract or a blank (ethanol). The mixtures were kept in a dark place for one hour, after which their absorbance was measured at 517 nm against the ethanolic blank.

The percentage of DPPH radical scavenging activity was calculated using Equation (1):

DPPH radical scavenging activity (%) = [1 − (A sample/A blank)] × 100(1)

where A sample represents the absorbance of the sample with the DPPH solution, and A blank is the absorbance of the DPPH solution without the sample.

Various sample concentrations (100–800 μg/mL) were tested to generate antiradical curves and determine EC_50_ values, which represent the concentration needed to scavenge 50% of free radicals. All measurements were conducted in triplicate.

The DPPH scavenging activity of *Artemisia*-ZnO NPs was assessed following the previously described method. A 1 mL aliquot of 0.1 mM DPPH methanolic solution was mixed with 1 mL of nanoparticle extract supernatant at various concentrations (50–400 μg/mL). The mixture was incubated in the dark for 30 min at room temperature, and the absorbance was measured at 517 nm. The percentage inhibition was calculated, and IC_50_ values (concentration required to inhibit 50% of DPPH radicals) were determined by plotting percentage inhibition against concentration.

#### 2.2.7. Antimicrobial Activity Assessment

The antimicrobial activity of the synthesized samples was evaluated against three reference pathogens obtained from the Microbiology Culture Collection of the Department of Biology, Gaziantep University: *Staphylococcus aureus* ATCC 25923 (Gram-positive, originally isolated from human skin lesions), *Escherichia coli* ATCC 25922 (Gram-negative, originally isolated from a human urinary tract infection), and *Candida albicans* ATCC 10231 (yeast, originally isolated from the human oral mucosa). These well-characterized standard strains are widely used in antimicrobial susceptibility testing due to their defined and reproducible response patterns. Bacterial cultures were maintained on nutrient agar slants at 4 °C, while the fungal strain was maintained on Sabouraud dextrose agar under similar conditions. Inocula were prepared by transferring a loopful of each test microorganism into nutrient broth (for bacteria) or Sabouraud broth (for fungi). Bacterial suspensions were incubated at 37 °C for 24 h, and fungal suspensions at 28 °C for 7 days, until moderate turbidity developed. The resulting suspensions were adjusted to match the 0.5 McFarland standard (~10^6^ CFU/mL) using sterile saline, ensuring consistency for antimicrobial testing.

##### Sample Preparation for Antimicrobial Testing

The ZnO nanoparticles were suspended in sterile distilled water at a concentration of 10 mg/mL and homogenized by sonication for 15 min to ensure even dispersion. The crude *A. absinthium* L. extract was dissolved in 5% DMSO to prepare a 10 mg/mL stock solution. Both stock solutions were sterilized using 0.22 µm syringe filters prior to use in assays.

##### The Minimum Inhibitory Concentration (MIC) and Minimum Bactericidal/Fungicidal Concentration (MBC/MFC)

MIC and MBC/MFC values were determined using the broth microdilution method in accordance with Clinical and Laboratory Standards Institute (CLSI) guidelines [17], with slight modifications. Serial two-fold dilutions of each test sample (ZnO NPs and plant extract) were prepared in sterile 96-well microplates using Mueller–Hinton broth (MHB) for bacterial strains and Sabouraud dextrose broth (SDB) for *Candida albicans*. The tested concentration range was 10.0, 5.0, 2.5, 1.25, 0.625, 0.3125, and 0.156 mg/mL. Each well received 100 μL of diluted test sample and 100 μL of microbial inoculum, previously standardized to the 0.5 McFarland standard (≈1 × 10^6^ CFU/mL), yielding a final volume of 200 μL per well. Negative control wells contained only broth and inoculum, while sterility controls included broth without inoculum or test sample. Plates were incubated at 37 °C for 24 h for bacterial strains and at 28 °C for 48 h for *C. albicans*. MIC was defined as the lowest concentration with no visible turbidity. For MBC/MFC determination, 10 µL aliquots from wells without visible growth were subcultured onto fresh agar plates (Mueller–Hinton or Sabouraud) and incubated again. MBC/MFC had the lowest concentration, yielding no colony formation.

##### Well Diffusion Method

Qualitative antimicrobial activity was further assessed using the agar well diffusion method [18]. Sterile Petri dishes were prepared with Mueller–Hinton agar for bacteria and Sabouraud dextrose agar for yeast. After solidification, the agar surface was inoculated with 100 µL of the microbial suspension (adjusted to 0.5 McFarland) spread evenly with a sterile swab. Wells of 6 mm diameter were bored aseptically into the agar, and 100 µL of test samples at concentrations from 0.1 to 10 mg/mL were added. Tetracycline (30 µg, Oxoid Ltd., Hampshire, UKwas used as the positive control for bacterial strains, while Fluconazole (25 µg, Oxoid Ltd., Hampshire, UK) served as the antifungal reference for *C. albicans*. Plates were incubated under appropriate conditions (37 °C/24 h for bacteria; 28 °C/48 h for yeast), and inhibition zones were measured in millimeters using a digital caliper. All antimicrobial results (zone diameters, MIC, and MBC/MFC values) were reported as mean ± standard deviation (SD) from three independent replicates.

#### 2.2.8. Statistical Analysis

Statistical differences between treatment groups were analyzed using one-way analysis of variance (ANOVA) followed by Tukey’s post hoc test for multiple comparisons. Differences were considered statistically significant at *p* < 0.05.

## 3. Results and Discussion

### 3.1. Phenolic Profile of A. absinthium *L.*

**Figure 1:**
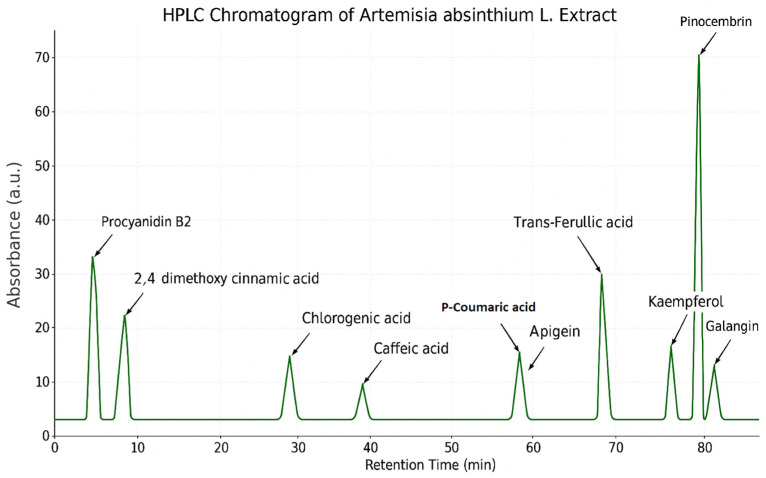
HPLC Chromatogram of *A. absinthium* L. Extract.

The chemical composition of *A. absinthium* L. was analyzed using HPLC, resulting in the identification of 12 phenolic compounds (Figure 1). These included six flavonoids-kaempferol, apigenin, galangin, pinocembrin, quercetin, and catechin, as well as several phenolic acids and other polyphenols, such as chlorogenic acid, caffeic acid, p-coumaric acid, trans-ferulic acid, 2,4-dimethoxycinnamic acid, and procyanidin B2 (Table 1). However, gallic acid was also detected, but could not be determined. Among all the compounds, pinocembrin had the highest concentration (69.16 ± 3.46 ppm), followed by procyanidin B2 (26.61 ± 1.33 ppm) and kaempferol (19.41 ± 0.97 ppm). In contrast, catechin and p-coumaric acid were present in minimal amounts, at 0.15 ± 0.01 ppm and 0.21 ± 0.01 ppm, respectively. These results suggest that *A. absinthium* L. is particularly rich in flavonoids with known antioxidant and antimicrobial properties [19]. These findings are consistent with the prior literature, in which caffeic acid, chlorogenic acid, quercetin, and ferulic acid have also been identified as major constituents of *Artemisia* species [6,20].

Moreover, the relatively high amounts of kaempferol and apigenin observed in this study support earlier reports suggesting that flavonoid biosynthesis is a key phytochemical feature of *A. absinthium* L. For example, studies on Tunisian and Iranian populations of *A. absinthium* L. have also noted substantial flavonoid presence, though concentrations vary due to climatic and geographic conditions [21,22,23]. This variation highlights the importance of environmental factors, extraction techniques, and plant maturity on phenolic profiles. The identified compounds in this study provide evidence of *A. absinthium* L.’s strong potential as a natural antioxidant source, supporting its traditional use and possible applications in food, pharmaceutical, and cosmetic industries [19,24,25].

### 3.2. Total Flavonoid Content, Total Phenolic Content, and Antioxidant Activity of A. absinthium *L.* Extract and Artemisia-ZnO NPs

In the current study, TFC of *A. absinthium* L. was determined to be 49.12 ± 0.04 mg quercetin equivalent per gram of extract (mg QE/g) (Table 2). In this study, 80% methanol was selected as the extraction solvent based on previous reports demonstrating its high efficacy in recovering flavonoid and phenolic compounds from *A. absinthium* L., while also allowing for comparability with existing literature data [26,27]. This result is consistent with a previous investigation that used 80% methanol as the extraction solvent, which reported a TFC of 49.01 ± 1.20 mg rutin equivalent per gram of dry extract (mg RE/g DE) [26]. On the other hand, a study employing aqueous extraction methods recorded a significantly lower TFC of 6.85 µg QE/mg of dry extract, emphasizing the substantial impact of solvent polarity on the efficiency of flavonoid extraction [19].

TPC of *A. absinthium* L. was found to be 119.45 ± 0.01 mg gallic acid equivalent per gram of extract (mg GAE/g) (Table 2). This value is lower than the findings of [27], who reported a higher TPC of 180.33 ± 16.25 mg GAE/g dry extract using unspecified extraction methods, suggesting variability due to methodological differences. In contrast, Kaoudoune et al. [28] recorded a lower TPC of 58.66 ± 2.16 mg GAE/g when aqueous extraction was employed, highlighting the influence of solvent type on phenolic yield.

The DPPH radical scavenging activity (IC_50_) of *A. absinthium* L. in the current study was found to be 369 µg/mL. A previous study reported a higher IC_50_ value of 612 ± 30.6 µg/mL for methanolic extracts of Iranian *A. absinthium* L. aerial parts at the flowering stage, indicating lower antioxidant activity compared to our findings [29]. In contrast, another study using aqueous extracts reported a significantly lower IC_50_ of 45.48 ± 0.37 µg/mL, demonstrating a stronger antioxidant potential [28]. The lower IC_50_ reflects the enhanced ability of the aqueous extract to neutralize DPPH radicals, demonstrating its greater antioxidant potency. Similarly, other investigations reported IC_50_ values of 85.23 µg/mL and 115.7 µg/mL for *A. absinthium* L. extracts obtained using ethanol and methanol, respectively, further highlighting the significant influence of solvent type and extraction method on antioxidant efficacy [23,27]. Variations in IC_50_ values reported across different studies may be attributed to multiple factors, including differences in extraction techniques, solvent types, and methods of plant preparation. Additionally, environmental conditions during plant growth and the maturation stage at the time of harvesting can significantly influence the phytochemical content and antioxidant activity of *A. absinthium* L.

The *A. absinthium* L. extract exhibited high levels of TFC and TPC, along with moderate antioxidant activity. After green synthesis at 80 °C, the resulting ZnO NPs retained measurable amounts of these bioactives, with a TPC of 52.34 ± 0.08 mg GAE/g and TFC of 18.65 ± 0.12 mg QE/g (Table 2). Notably, the DPPH radical scavenging activity was enhanced in the ZnO NPs (IC_50_ = 211 ± 0.05 μg/mL), suggesting synergistic effects between the nanoparticle surface and plant-derived functional groups. The results in Table 2 reveal significant differences (*p* < 0.05) between the crude *A. absinthium* L. extract and the biosynthesized ZnO nanoparticles in terms of TFC, TPC, and antioxidant activity. While both total phenolic and flavonoid contents were substantially reduced in the nanoparticle formulation—likely due to heat-induced degradation or limited surface adsorption—a considerable amount of bioactive compounds was still retained. Notably, the DPPH IC_50_ value of the ZnO NPs was significantly lower than that of the extract, indicating enhanced antioxidant activity. This suggests a possible synergistic effect between the residual phytochemicals and the inherent ROS-scavenging property of ZnO nanoparticles, supporting their potential use in biomedical and food preservation applications.

### 3.3. Characterization of Artemisia-ZnO NPs

The size and morphology of ZnO NPs are critically influenced by the synthesis parameters employed during their production. Key factors such as precursor concentration, solution pH, reaction temperature, reaction time, and post-synthesis treatments like calcination or annealing play important roles in determining the physical and chemical characteristics of the final nanomaterial [30,31]. For example, precursor concentration directly affects the supersaturation level of Zn^2+^ ions, which controls the nucleation rate and, consequently, the final particle size. A higher precursor concentration often leads to larger particles due to faster nucleation and growth rates [31]. Meanwhile, the pH of the reaction medium is known to influence the ionization and solubility of zinc salts and phytochemicals involved in green synthesis. Lower pH values (acidic conditions) typically result in the formation of spherical and more uniform particles, while higher pH (alkaline conditions) can cause aggregation or elongated morphologies, depending on the stability of intermediate complexes formed during synthesis [32]. Reaction temperature is another decisive factor: at moderate temperatures (50–80 °C), ZnO nanoparticles tend to exhibit a relatively spherical or ellipsoidal shape, especially in biosynthetic approaches. However, when the temperature is elevated (above 100 °C), the increased kinetic energy promotes anisotropic crystal growth, often resulting in rod-shaped, needle-like, or even flower-like structures. This is primarily due to the preferential growth of certain ZnO crystal planes under thermal acceleration [33]. Furthermore, calcination duration and temperature during the final drying or post-synthesis treatment phase significantly affect both the crystallinity and surface area of the ZnO NPs. Prolonged calcination at high temperatures (e.g., 400–600 °C) typically enhances crystallinity but may also induce particle agglomeration or grain growth, leading to an increase in average particle size and a loss in surface reactivity [34]. In green synthesis specifically, where plant extracts are used as reducing and stabilizing agents, these parameters must also be considered in the context of phytochemical stability. Excessive heat or extremely alkaline conditions can degrade flavonoids, phenolics, and other active compounds responsible for the reduction of Zn^2+^ and capping of the nanoparticles [33].

Several studies have investigated the green synthesis of ZnO NPs using various *Artemisia* species, revealing notable differences in particle size and morphology depending on the specific plant type and synthesis conditions (Table 3). For instance, one previous study synthesized ZnO nanoparticles using *Artemisia abyssinica* leaf extract and reported particle sizes ranging from 5 to 22 nm with a nearly spherical morphology, as confirmed by scanning electron microscopy (SEM) [35]. Similarly, another study utilized *Artemisia annua* for nanoparticle synthesis and observed spherical particles measuring approximately from 21.34 to 24.71 nm [36]. Interestingly, nanorod-shaped ZnO NPs with a larger average particle size of 50.29 nm were synthesized using *Artemisia abrotanum*, suggesting that different phytochemical compositions may influence crystal growth directions [4]. Additionally, spherical ZnO NPs with an average size of 17.82 nm were produced from *Artemisia vulgaris* [37], while another study reported highly uniform spherical nanoparticles of approximately 17 nm synthesized using an unspecified *Artemisia* species [38]. These findings demonstrate that *Artemisia*-mediated green synthesis routes can produce ZnO NPs with controlled size and shape, depending on factors such as plant species, extraction method, and post-synthesis treatment.

**Figure 2:**
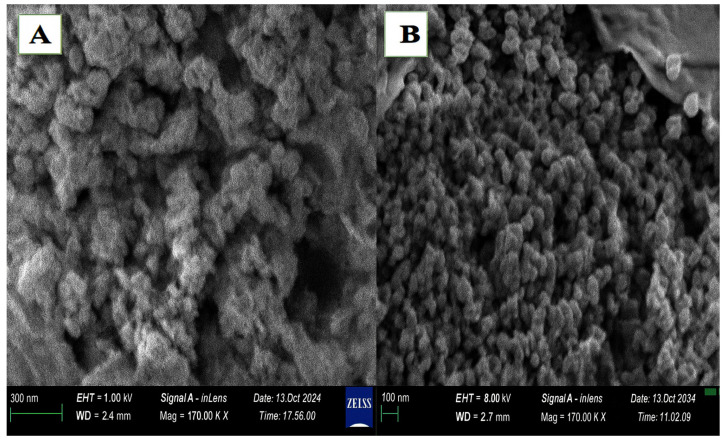
FE-SEM images show that the particles have a nanostructured, spherical, and granular appearance. (**A**) is *Artemisia*-ZnO NPs, and (**B**) is ZnO NPs.

The FE-SEM image in Figure 2 shows that the synthesized nanoparticles possess a spherical and granular nanostructure. The morphology of the *Artemisia*-ZnO NPs exhibited minimal agglomeration, with an average size of approximately 36.6 nm. In contrast, the pure ZnO NPs (without *A. absinthium* L.) showed an average diameter of 28.7 nm. Both nanoparticle types were uniformly distributed across the carbon-coated copper grid, confirming the successful formation of fine, nanoscale particles within the desired size range. The smaller average particle size of pure ZnO NPs compared to *Artemisia*-ZnO NPs could be attributed to the influence of phytochemicals present in the *A. absinthium* L. extract. These organic compounds act as capping agents, promoting particle stabilization but potentially leading to larger particle sizes due to molecular interactions and surface modifications. In contrast, pure ZnO NPs, synthesized without plant extract, may form more compact and smaller particles due to the absence of such stabilizing agents, allowing for more controlled nucleation and growth [39]. Phytochemicals not only act as capping agents but also enhance the properties of ZnO NPs. A previous study highlighted that green synthesis using plant extracts resulted in ZnO NPs with better properties compared to those synthesized chemically, indicating the role of phytochemicals in controlling the size and enhancing functionality [40]. Similarly, they found that the choice of capping agent significantly influences the particle size of ZnO NPs. For instance, using soluble starch as a capping agent resulted in smaller particle sizes (3–5 nm), whereas other agents led to larger sizes [40]. Another study synthesized ZnO NPs using leaf extracts from different plants and observed variations in particle sizes. ZnO NPs produced by *Olea europaea* extract had average sizes of 48.2 nm, while those synthesized with *Matricaria chamomilla* extract were larger. This suggests that different phytochemicals in plant extracts can influence the size of ZnO NPs [31].

**Figure 3:**
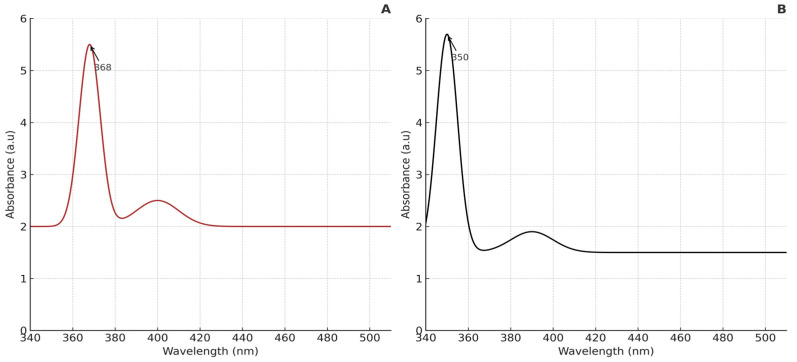
UV–Visible spectrum of the synthesized ZnO NPs. (**A**) *Artemisia*-ZnO NPs (**B**) Pure ZnO NPs.

The UV–visible absorption spectrum of the synthesized nanoparticles is displayed in Figure 3. A prominent peak around 368 nm is observed, which is characteristic of ZnO NPs and corresponds to their high excitation binding energy at room temperature [41]. It can be seen from Figure 3 that there was intensive absorption in the ultraviolet band of about 200–400 nm. The absorption wavelength at about 368 nm of *Artemisia*-ZnO NPs suggested the excitonic character at room temperature. For pure ZnO NPs (without *A. absinthium* L.), absorption typically occurred around 350 nm. These results are consistent with findings from previous studies [15,42].

**Figure 4:**
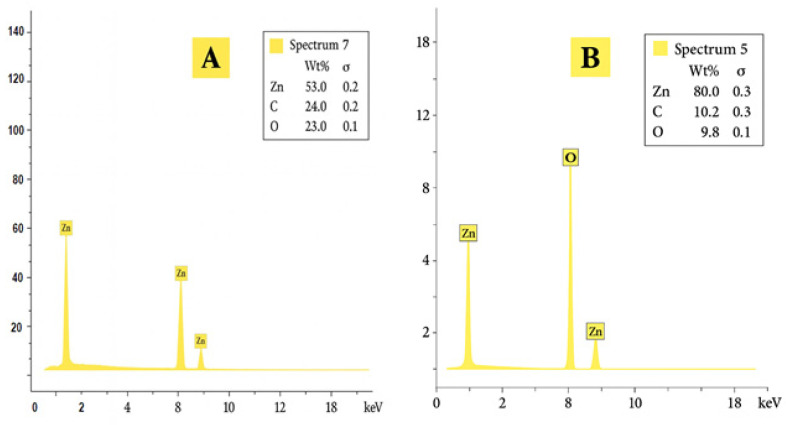
EDX spectra of zinc oxide nanoparticles, comparing (**A**) *Artemisia*-ZnO NPs and (**B**) Pure ZnO NPs.

The EDX analysis of pure ZnO NPs without *A. absinthium* L. indicates that zinc is the predominant element, with no significant impurities detected (Figure 4). The elemental composition reveals that zinc constitutes approximately 80.0%, oxygen 9.8%, and carbon 10.2%, respectively, by weight of the pure nanoparticles. Similarly, the result of *Artemisia*-ZnO NPs contained 53% zinc, 23% oxygen, and 24% carbon by weight. The notably higher carbon content in the *Artemisia*-ZnO NPs can be explained by the presence of organic compounds in the *A. absinthium* L. extract. These phytochemicals—such as polyphenols, flavonoids, and other bioactive molecules—act as capping agents during nanoparticle synthesis. They not only stabilize the nanoparticles and prevent agglomeration but also remain attached to the nanoparticle surface, thereby increasing the carbon content detected by EDX [43,44]. This finding aligns with observations from other studies on biosynthesized ZnO NPs. For instance, a previous study reported that ZnO NPs synthesized using *A. absinthium stelleriana* as a capping agent primarily contained zinc, oxygen, and carbon, which corroborates the influence of plant-derived organic residues on the elemental composition of ZnO NPs [45]. In another study, ZnO NPs synthesized using *Cayratia pedata* leaf extract showed zinc and oxygen percentages of 78.32% and 12.78%, respectively—values higher in zinc content and lower in organic content compared to the *Artemisia*-mediated synthesis [43]. Additionally, another report demonstrated that ZnO NPs produced with *Moringa oleifera* seed extract had an elemental composition of 86.79% zinc, 10.48% oxygen, and only 2.73% carbon. This indicates that the type of plant extract and the synthesis conditions significantly affect the final nanoparticle composition [44].

**Figure 5:**
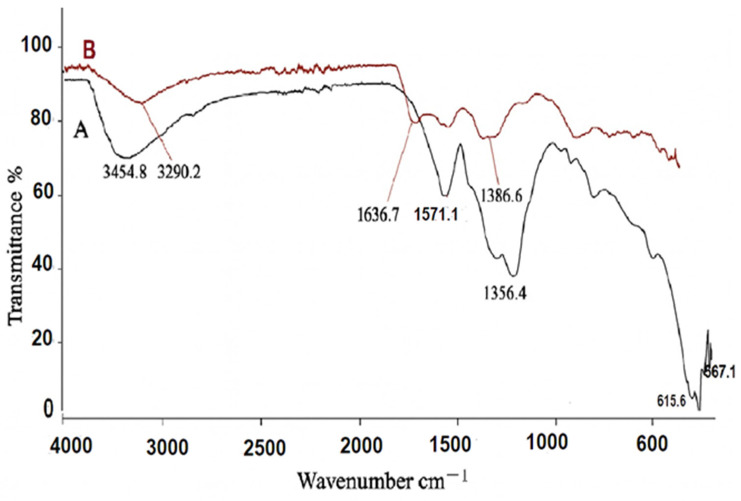
FT-IR spectra of (A) *Artemisia*-ZnO NPs (black line) and (B) pure *A. absinthium* L. extract (red line).

The FT-IR analysis of the synthesized nanoparticles is presented in Figure 5. This infrared study was conducted to confirm the purity and composition of the nanoparticles, as well as to identify the presence of phytochemicals from the extract. Various phytochemicals, including alcohols, phenols, amines, and carboxylic acids, can interact with the zinc surface, playing a crucial role in the stabilization of ZnO NPs. The FT-IR spectrum of pure ZnO NPs primarily exhibits characteristic peaks in the 600–400 cm^−1^ range, corresponding to Zn–O stretching vibrations, confirming the formation of ZnO bonds [39,46]. In comparison, the FT-IR spectrum of *Artemisia*-ZnO NPs includes Zn–O stretching peaks at 615 cm^−1^ and 567cm^−1^, which align with metal oxide vibrations but also feature additional peaks from phytochemical compounds. The broad peak around 3454 cm^−1^ suggests the presence of hydroxyl (-OH) groups, likely from flavonoids or polyphenols in *A. absinthium* L. extract, while the peak at 1571 cm^−1^ corresponds to C=C stretching, indicative of organic molecules interacting with the nanoparticle surface. These differences confirm that *A. absinthium* L. extract acts as a capping and stabilizing agent in the green synthesis of ZnO NPs, leading to structural and chemical modifications compared to pure ZnO nanoparticles [31,45,47]. Furthermore, the spectral peaks observed in this study align with previous findings, reinforcing the role of plant-based compounds in nanoparticle synthesis and stabilization [15,42,45].

### 3.4. Antimicrobial Activity Results

The comparative analysis of MIC and MBC/MFC values for *Artemisia*-ZnO NPs, pure ZnO NPs, and *A. absinthium* L. extract alone against *S. aureus*, *E. coli*, and *C. albicans* reveals significant differences in antimicrobial efficacy (Table 4).

The MIC and MBC/MFC values for *Artemisia*-ZnO NPs are notably lower across all tested microorganisms compared to pure ZnO NPs and *A. absinthium* L. extract alone, indicating enhanced antimicrobial activity. For instance, against *S. aureus*, the MIC for *Artemisia*-ZnO NPs is 0.312 mg/mL, whereas both pure ZnO NPs and *A. absinthium* L. extract exhibit MICs of 0.625 mg/mL. Similarly, the MIC for *E. coli* is 1.25 mg/mL for *Artemisia*-ZnO NPs, compared to 2.50 mg/mL for the other two agents. In the case of *C. albicans*, *Artemisia*-ZnO NPs demonstrate a MIC of 0.625 mg/mL, while pure ZnO NPs and *A. absinthium* L. extract both have MICs of 2.50 mg/mL. These findings suggest that the biosynthesis of ZnO NPs using *A. absinthium* L. extract enhances antimicrobial efficacy, potentially due to the synergistic effects of ZnO and the phytochemicals present in the plant extract. This is consistent with previous studies indicating that green-synthesized ZnO NPs exhibit superior antimicrobial properties compared to their chemically synthesized counterparts. For example, a previous study reported that green-synthesized ZnO NPs had enhanced antibacterial activity against both Gram-positive and Gram-negative bacteria compared to pure ZnO NPs [18]. The enhanced antifungal efficacy of *Artemisia*-ZnO NPs against *C. albicans* observed in the current study is consistent with findings from other plant-mediated ZnO NPs studies. One such study demonstrated that ZnO NPs synthesized using plant extracts showed significantly lower MIC values against *C. albicans* compared to pure ZnO NPs [48]. These results clearly demonstrate that the incorporation of *A. absinthium* L. extract in the green synthesis of ZnO NPs significantly enhances their antimicrobial activity against *S. aureus*, *E. coli*, and *C. albicans*, compared to pure ZnO NPs and the plant extract alone. This enhancement is likely due to the synergistic effects between ZnO and the bioactive compounds in *A. absinthium* L., leading to more effective antimicrobial agents.

A recent study reported the biosynthesis of ZnO NPs using *Artemisia afra* extracts, where the resulting NPs demonstrated enhanced antibacterial activity against *E. coli* and *S. aureus*. This improvement was attributed to synergistic effects between ZnO and the phytochemicals present in the *Artemisia* extract, which served as capping agents, contributing to the nanoparticles’ stability and bioactivity [49]. Similarly, ZnO NPs synthesized with *Artemisia annua* extracts showed significant antimicrobial properties and promoted osteogenic differentiation in human osteoblast-like cells, further supporting the role of plant-based bioactives in enhancing nanoparticle functionality [50]. Although direct studies on *Artemisia*-ZnO NPs are limited, the consistent findings across different *Artemisia* species suggest the potential for similar synergistic effects. Owing to the rich content of phenolic compounds, ZnO NPs synthesized with its extract are likely to exhibit enhanced antimicrobial properties through synergistic interactions.

The results of Table 5 show that *Artemisia*-ZnO NPs exhibited enhanced inhibitory effects against all tested microorganisms when compared to both pure ZnO NPs and *A. absinthium* L. extract alone. Statistically significant differences (*p* < 0.05) were observed in most comparisons. Against *E. coli*, the *A. absinthium* L. extract displayed the largest inhibition zone (18.00 ± 5.00 mm), followed by the *Artemisia*-ZnO NPs (14.00 ± 2.56 mm), with pure ZnO NPs showing the lowest activity (9.00 ± 0.25 mm). Interestingly, the positive control, Tetracycline (30 µg), produced a zone of 16.00 ± 3.75 mm, which was comparable to the extract and higher than the nanoparticle formulations. This suggests that phytochemical-rich extracts of *A. absinthium* L. may exhibit antimicrobial activity comparable to conventional antibiotics in some cases. For *C. albicans*, the *Artemisia*-ZnO NPs (18.00 ± 3.43 mm) and *A. absinthium* L. extract (16.00 ± 2.83 mm) both significantly surpassed pure ZnO NPs (10.00 ± 0.17 mm), but remained less effective than the positive control, Fluconazole (25 µg), which showed the largest zone of inhibition at 25.00 ± 4.30 mm. This result supports previous findings [51] that reported antifungal activity of chemically synthesized ZnO NPs, and suggests that capping with *A. absinthium* L. enhances antifungal efficacy. In the case of *S. aureus*, the *Artemisia*-ZnO NPs showed the highest antimicrobial activity among the test samples (24.00 ± 4.00 mm), followed by the *A. absinthium* L. extract (22.28 ± 6.51 mm) and pure ZnO NPs (12.00 ± 1.38 mm). The positive control, Tetracycline, showed the largest inhibition zone (30.00 ± 7.88 mm), confirming its superior efficacy. Nonetheless, the *Artemisia*-based treatments demonstrated potent antimicrobial activity, especially when incorporated into ZnO nanostructures. These results are consistent with earlier studies [52,53], which reported that Gram-positive bacteria tend to be more susceptible to ZnO NPs, likely due to structural differences in their cell walls that facilitate nanoparticle penetration and disruption. Importantly, this is among the first studies to report the combined antimicrobial effect of *A. absinthium* L. phytochemicals and ZnO NPs, showing a clear synergistic enhancement in activity, particularly against *S. aureus* and *C. albicans*. While other *Artemisia* species (e.g., *A. afra* and *A. annua*) have shown similar effects in previous studies like [49,50], no prior study to our knowledge has quantified this effect using *A. absinthium* L. in a well diffusion method.

## 4. Conclusions

This study successfully demonstrated the green synthesis of ZnO NPs using *A. absinthium* L. extract, highlighting their superior physicochemical characteristics and significantly enhanced antimicrobial activity. The synthesized *Artemisia*-ZnO NPs exhibited spherical morphology with an average particle size of 36.6 nm and strong absorption features consistent with ZnO nanostructures. Phytochemical analysis revealed the presence of potent reducing and stabilizing agents, which contributed not only to effective nanoparticle formation but also to their antimicrobial functionality. The *Artemisia*-ZnO NPs showed significantly higher antimicrobial activity against *S. aureus*, *E. coli*, and *C. albicans* compared to the extract and pure ZnO NPs, supported by MIC, MBC/MFC, and inhibition zone data. Additionally, they retained measurable levels of TPC (52.34 mg GAE/g) and TFC (18.65 mg QE/g), with enhanced DPPH radical scavenging activity (IC_50_ = 211 μg/mL), suggesting synergistic effects between ZnO and residual bioactives. Notably, this study is among the first to systematically compare the antimicrobial efficacy of *Artemisia*-ZnO NPs, the plant extract, and pure ZnO NPs using both broth microdilution and well diffusion methods against multiple microorganisms. The findings contribute novel insights to the expanding field of green nanotechnology and underscore the potential of *A. absinthium* L. as an effective biogenic mediator for the development of functional nanomaterials. Given their strong antibacterial and antifungal activity, the synthesized *Artemisia*-ZnO NPs demonstrate significant promise for applications in pharmaceutical, food safety, and biomedical sectors as eco-friendly and biologically active agents. However, further in vivo studies and cytotoxicity assessments are necessary to comprehensively evaluate their clinical applicability.

## Figures and Tables

**Table 1:** Phenolic content of *A. absinthium* L.

Phenolic Compounds	RET. Time	Quantity [ppm]	R^2^
Chlorogenic acid	33.268	6.12 ± 0.31	0.9965
Caffeic acid	38.098	4.49 ± 0.22	0.9978
Apigenin	62.415	10.01 ± 0.50	0.9923
P-Coumaric acid	57.785	0.21 ± 0.01	0.997
Galangin	78.619	3.12 ± 0.16	0.9916
Trans-Ferulic acid	62.275	9.68 ± 0.48	0.9955
Kaempferol	75.474	19.41 ± 0.97	0.9944
2,4-dimethoxy cinnamic acid	5.001	11.87 ± 0.59	0.9957
Pinocembrin	78.146	69.16 ± 3.46	0.9999
Procyanidin B2	2.960	26.61 ± 1.33	0.9965
Quercetin	75.818	0.88 ± 0.04	0.9989
Catechin	41.270	0.15 ± 0.01	0.9995

**Table 2:** Total flavonoid content (TFC), total phenolic content (TPC), and DPPH radical scavenging activity of *A. absinthium* L. extract and ZnO nanoparticles synthesized at 80 °C.

Parameter	Unit	*A. absinthium* L. Extract	*Artemisia*-ZnO NPs (80 °C Synthesis)
Total Flavonoid Content	mg QE/g	49.12 ± 0.04 ^a^	18.65 ± 0.12 ^b^
Total Phenolic Content	mgGAE/g	119.45 ± 0.01 ^a^	52.34 ± 0.08 ^b^
DPPH	μg/mL	369 ± 0.03 ^a^	211 ± 0.05 ^b^

The mg QE/g is mg quercetin equivalent per gram *A. absinthium* L., and mg GAE/g is mg gallic acid equivalent per gram *A. absinthium* L. Values are expressed as mean ± SD (*n* = 3). Different superscript letters in the same row indicate statistically significant differences (*p* < 0.05).

**Table 3:** A comparative table summarizing studies on the green synthesis of ZnO NPs using various *Artemisia* species, focusing on specific particle sizes and morphologies.

Study	Plant Species	Particle Size [nm]	Morphology	Synthesis Method
Galedari & Teimouri (2020) [4]	*Artemisia* sp.	17	Spherical	Green synthesis using plant extract; characterized by SEM and TEM
Orshiso & Zereffa (2023) [35]	*Artemisia abyssinica*	5–22	Nearly spherical	Green synthesis using leaf extract; characterized by SEM
Sesime et al., (2021) [36]	*Artemisia annua*	21.34–24.71	Spherical	Green synthesis using plant extract; characterized by XRD and SEM
Azeez et al., (2024) [38]	*Artemisia abrotanum*	50.29	Nanorod	Green synthesis using leaf extract; characterized by SEM
Acharya et al., (2024) [37]	*Artemisia vulgaris*	17.82	Spherical	Green synthesis using leaf extract; characterized by SEM

**Table 4:** Minimum inhibitory concentration (MIC) and minimum cidal concentration (MBC/MFC) of *Artemisia*-ZnO NPs, Pure ZnO, and Extract on *S. aureus*, *E. coli*, and *C. albicans*.

Microorganism	Agent	MIC [mg/mL]	MBC [mg/mL]
*S. aureus*	*Artemisia*-ZnO NPs	0.312 ± 0.00 ^a^	0.625 ± 0.00 ^a^
	Pure ZnO [uncapped]	0.625 ± 0.00 ^b^	1.25 ± 0.00 ^b^
	*A. absinthium* L. extract	0.625 ± 0.00 ^b^	1.25 ± 0.00 ^b^
*E. coli*	*Artemisia*-ZnO NPs	1.25 ± 0.00 ^a^	2.50 ± 0.00 ^a^
	Pure ZnO NPs	2.50 ± 0.00 ^b^	2.50 ± 0.00 ^a^
	*A. absinthium* L. extract	2.50 ± 0.00 ^b^	5.00 ± 0.00 ^b^
*C. albicans*	*Artemisia*-ZnO NPs	0.625 ± 0.00 ^a^	1.25 ± 0.00 ^a^
	Pure ZnO NPs	2.50 ± 0.00 ^b^	5.00 ± 0.00 ^b^
	*A. absinthium* L. extract	2.50 ± 0.00 ^b^	5.00 ± 0.00 ^b^

Values are mean ± SD; different superscript letters within each row indicate statistically significant differences, *p* < 0.05.

**Table 5:** Inhibition Zone Diameters (mm) of Pure ZnO NPs, *A. absinthium* L. Extract, and *Artemisia*-ZnO NPs against Test Microorganisms.

Microorganism	Pure ZnO NPs	*A. absinthium* L. Extract	*Artemisia*-ZnO NPs	Positive Control
*E. coli*	9.00 ± 0.25 ^c^	18.00 ± 5.00 ^a^	14.00 ± 2.56 ^b^	16.00 ± 3.75 ^ab^ (Tetracycline)
*C.* *albicans*	10.00 ± 0.17 ^c^	16.00 ± 2.83 ^b^	18.00 ± 3.43 ^b^	25.00 ± 4.30 ^a^ (Fluconazole)
*S. aureus*	12.00 ± 1.38 ^c^	22.28 ± 6.51 ^b^	24.00 ± 4.00 ^a^	30.00 ± 7.88 ^a^ (Tetracyclin)

Values are mean ± SD; different superscript letters within each row indicate statistically significant differences, *p* < 0.05.

## Data Availability

The data generated and/or analyzed during the current study are available from the corresponding author upon reasonable request.
